# Surgical exploration without resection in pancreatic and periampullary tumors: Report from a national database

**DOI:** 10.1177/1457496920913669

**Published:** 2020-04-17

**Authors:** Emil Sahlström, Johan Nilsson, Bobby Tingstedt, Magnus Bergenfeldt, Roland Andersson, Bodil Andersson

**Affiliations:** Department of Clinical Sciences, Lund, Surgery, Lund University and Skåne University Hospital, Lund, Sweden; Department of Clinical Sciences, Lund, Cardiothoracic Surgery Lund, Lund University and Skåne University Hospital, Lund, Sweden; Department of Clinical Sciences, Lund, Surgery, Lund University and Skåne University Hospital, Lund, Sweden; Department of Clinical Sciences, Lund, Surgery, Lund University and Skåne University Hospital, Lund, Sweden; Department of Clinical Sciences, Lund, Surgery, Lund University and Skåne University Hospital, Lund, Sweden; Department of Surgery, Clinical Sciences, Lund, Lund University and Skåne University Hospital, Lund, SE-221 85, Sweden

**Keywords:** Pancreatic cancer, periampullary cancer, pancreatic resection, palliative surgery, unresectability, survival

## Abstract

**Background and objective:**

Pancreatic and periampullary cancers are sometimes found to have a too advanced disease during surgery to allow resection. The aim was to describe characteristics, treatment, outcome, and time trends for patients that were planned for pancreatic surgery but found unresectable during surgery.

**Methods:**

Data from the Swedish National Pancreatic and Periampullary Cancer Registry were used. All patients registered between January 2010 and August 2018 were included. The patient cohort was divided in two halves based on year of diagnosis.

**Results:**

In total, 12,377 patients were included in the registry and finally 4568 patients were scheduled for surgery. During surgical exploration, 3879 (84.9%) patients underwent pancreatic resection, 658 (14.4%) patients were found unresectable, and 31 (0.7%) had no pancreatic resection due to other reasons (e.g. benign lesion, comorbidity). More patients underwent surgical exploration and resection during the second time period, but exploration without resection was unchanged (15.7% vs 13.7%; p = 0.062). Survival rates were lower among the unresectable patients with pancreatic and periampullary tumors compared to the resectable patients, including 30-day mortality (n = 17 (3.5%) vs n = 39 (1.6%), p = 0.004) and 90-day mortality (n = 72 (15.0%) vs n = 70 (2.8%), p < 0.001). Palliative surgery became less common during the second half of the time period (p < 0.001).

**Conclusions:**

Unresectability is associated with an unfavorable prognosis. The frequency did not decrease during the study period, but palliative surgical procedures became less common.

## Introduction

Surgery is the only curative option for patients with pancreatic and periampullary cancer since the majority of the tumors will progress despite chemotherapy and radiotherapy^1^. Unfortunately, many of these tumors are found unresectable at the time of diagnosis since symptoms occur late in the progression of the disease. Only approximately 20%–25% of all patients diagnosed with pancreatic cancer are resectable^2^. The surgical resection is however extensive and associated with complications, why patient selection must be done carefully^3^.

The predicted resectability of a tumor in pancreas or the periampullary region is primarily based on the results of a computed tomography (CT)^4^. Unfortunately, the diagnostic accuracy of CT is low, and tumor spread is often underestimated. This is problematic since unnecessary laparotomy results in morbidity for the patient and increased costs. It also delays the start of palliative treatment. Around 10%–25% of all patients with pancreatic cancer undergoing potentially curative surgery are found to have an unresectable lesion during the operation^4^.

When a presumed resectable tumor turns out to be locally advanced or metastasized perioperatively, the option is to do nothing further or to perform endoscopy or palliative surgery, for example, hepaticojejunostomy and/or gastrojejunostomy (double bypass). The double bypass has earlier been considered somewhat of a standard treatment. However, recent studies have questioned the use of additional surgery in a palliative situation, suggesting a more conservative approach. This conclusion was motivated by the fact that perioperative palliative surgery did not prevent future complications. Patients who underwent a double bypass also experienced more severe complications, had longer hospital stay, and the start of palliative chemotherapy was delayed^5,6^.

The primary aim of this register study, using data from the Swedish National Pancreatic and Periampullary Cancer Registry, was to describe characteristics and outcome of all patients subjected to pancreatic surgery, with focus on patients that were found unresectable during surgical exploration, and to investigate if the surgery and resection rates have changed over time. The secondary aim was to evaluate the use and result of palliative surgery.

## Material and Methods

### Patients and Data Collection

The study was based on data from the Swedish National Pancreatic and Periampullary Cancer Registry, which is a multicenter, nationwide, non-selected cohort. The registry consists of six forms and contains both preoperative, perioperative, and postoperative information about the patients. A complete list of variables is available on the web page of the register^7^. National and regional data are analyzed, and the results are e-published once a year^8^.

All Swedish patients with a suspected malignant pancreatic lesion and all patients subjected to pancreatic surgery are included in the registry. The registry was established in 2010, and its validity and coverage has shown good results^9^. Register data were received up to 28 August 2018.

All patients registered between January 2010 until August 2018 were included. The patients were included regardless of the result of the histopathology. An unresectable tumor was defined as a locally advanced primary tumor, not possible to resect, or a metastasized disease. The tumor was considered locally advanced in the presence of non-resectable extensive venous tumor invasion (i.e. extensive invasion of the portal vein or the superior mesenteric vein) and/or non-resectable arterial tumor invasion (i.e. invasion of the superior mesenteric artery, the celiac axis, or the hepatic artery)^6,10^. Palliative surgery performed instead of radical surgery during the planned operation was recorded and analyzed.

For the survival analysis, only patients with histologically proven pancreatic and periampullary cancer were included (excluding endocrine tumors, benign lesions, chronic pancreatitis, metastasis from other cancers, unknown histopathology, see Table 1). Also, patients that underwent other resections (e.g. endoscopic polypectomy) and patients in a poor condition during surgery disabling resection (e.g. portal hypertension) were excluded.

**Table 1.** Histopathology of the patients undergoing pancreatic surgery, in relation to if the tumor was resected or not.

Variable name	n	Unresectable tumor (n = 658)	Resectable tumor (n = 3879)
Pancreatic cancer	2133	423 (64.3%)	1710 (44.1%)
Distal cholangiocarcinoma	273	26 (4.0%)	247 (6.4%)
Duodenal cancer	238	19 (2.9%)	219 (5.6%)
Ampullary cancer	300	13 (2.0%)	287 (7.4%)
Endocrine cancer	170	5 (0.8%)	165 (4.3%)
Metastasis from other cancer	75	19 (2.9%)	56 (1.4%)
Benign lesion	605	5 (0.8%)	600 (15.5%)
Chronic pancreatitis	153	9 (1.4%)	144 (3.7%)
Other/unknown lesion	590	139 (21.1%)	451 (11.6%)

Data are presented as absolute numbers (percentage).

The patients were divided in two based on whether he or she was diagnosed before or after 31 December 2013.

### Statistics

Data are presented as means with standard deviations (SDs) and medians with interquartile range (IQR). Baseline characteristics between patients with resectable and unresectable disease were compared using the Student’s t-test or the unpaired Mann–Whitney U test for continuous variables, and chi-square-test for categorical variables. The Kaplan–Meier estimate of the survivor function was used to estimate long-term survival. The log-rank test was used to compare survival difference between the groups.

All statistical analyses were made two-sided. A p-value <0.05 was considered significant. Statistical analysis and graphs were performed using Stata MP statistical package version 15.1, 2017 (StataCorp LP, College Station, TX, United States). Ethics were approved by the Regional Human Ethics Committee at Lund University (Dnr 2018/499).

## Results

**Fig. 1.**
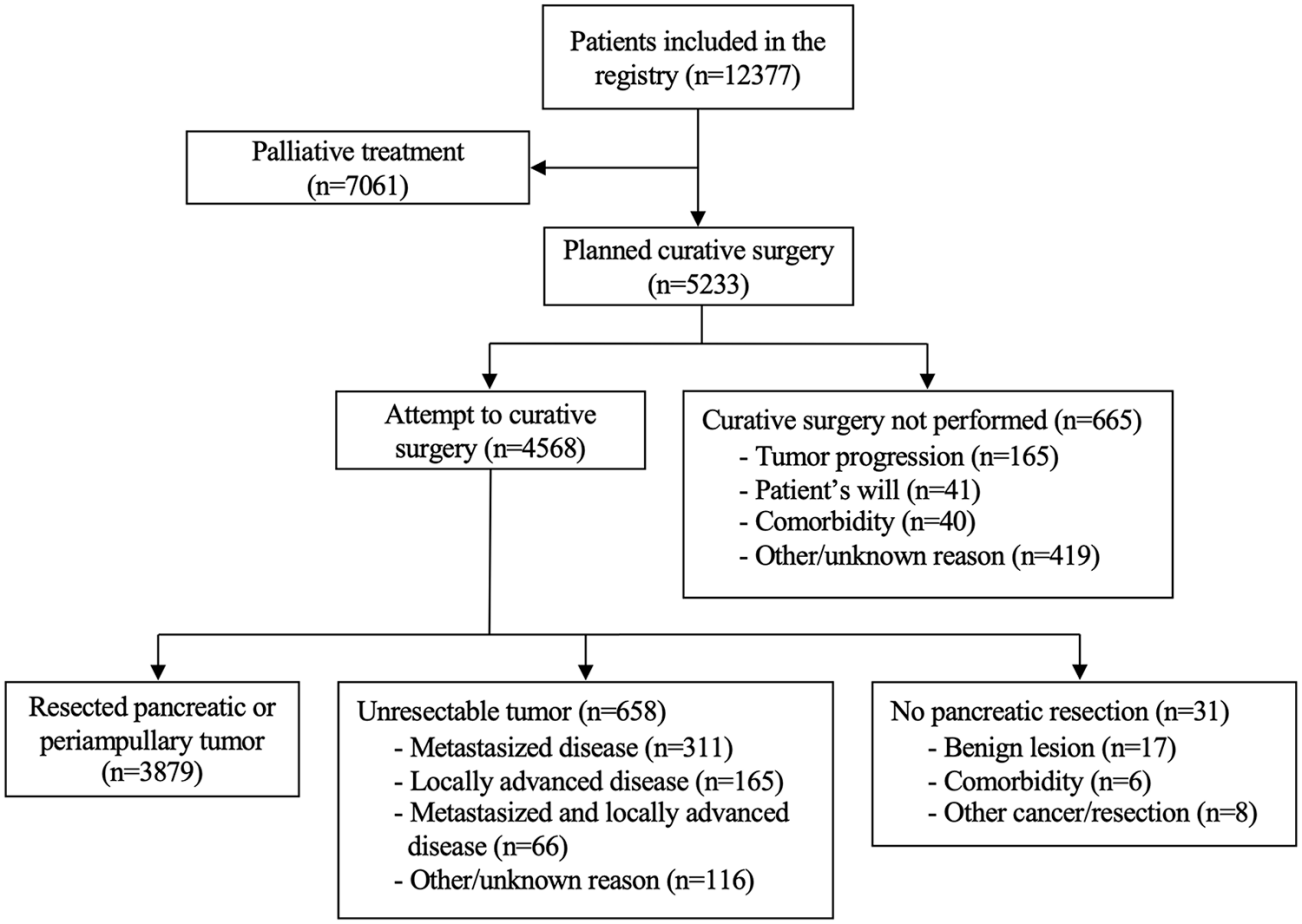
Flow chart describing the sequence of events for all patients included in the Swedish National Pancreatic and Periampullary Cancer Registry.

In total, 12,377 patients were included in the Swedish National Pancreatic and Periampullary Cancer Registry. Gender distribution was even, 6292 patients (50.8%) were male and 6085 patients (49.2%) were female. Overall mean age at diagnosis was 69.4 ± 11.1 years, and the male patients were younger (68.9 ± 10.6 vs 69.9 ± 11.5 years, p < 0.001). At the time of diagnosis, 7061 patients (57.4%) were considered palliative and 5233 patients (42.6%) were planned for curative treatment (i.e. resection). Finally, 4568 of these patients (87.3%) underwent surgery. The number one reason for not performing surgery (n = 665) in the group planned for intervention was tumor progression, followed by will of the patient and comorbidity (Fig. 1). Neoadjuvant chemotherapy was administered to 190 patients (4.2%) who later underwent surgical exploration, with resection in 140 (73.7%) patients. More patients underwent exploration (with or without resection) during the second time period, even if no change in the proportion of patients subjected to surgery was seen when comparing all patients in the registry (n = 1771 vs n = 2797, p = 0.264).

During surgery, 3879 patients (84.9%) were considered resectable, 658 patients (14.4%) were not resected due to a locally advanced or metastasized tumor situation, and 31 patients (0.7%) had no pancreatic resection due to benign disease, other malignant disease, other resection, or intraoperative too poor medical condition to allow resection (Fig. 1). More patients underwent resection during the second time period, but as for exploration no increase could be seen in the proportion when comparing with all patients (n = 1484 vs n = 2395, p = 0.062). When a tumor was deemed unresectable, the underlying cause was more often a metastasized disease than a locally advanced tumor (312 vs 169). In several cases, both a metastasized and locally advanced disease were found (Fig. 1).

The final result of the histopathology of the patients undergoing surgery, based on if the tumor was resected or not, is presented in Table 1. In the group that underwent pancreatic surgery, 15.5% (n = 600) were found to have a benign tumor, and in the unresectable group, 0.9% (n = 6) had a benign tumor.

Preoperative variables, in relation to if the disease was resected or not, are presented in Table 2. Several factors are as expected more common in unresectable patients, for example, involuntary weight loss, older age, higher C-reactive protein (CRP), and cancer antigen (CA) 19-9.

**Table 2:** Preoperative characteristics of the patient group with an unresectable tumor in comparison to the patient group with a resectable tumor.

Variable name	n	Unresectable tumor (n = 658)	Resectable tumor (n = 3879)	p-value
Male gender	4537	345 (52.4%)	2013 (51.9%)	0.799
Presence of involuntary weight loss	4435	433 (65.8%)	1624 (41.9%)	<0.001
Diabetes mellitus	4463	187 (28.4%)	749 (19.3%)	<0.001
Smoking	4347	106 (16.1%)	597 (15.4%)	0.672
Preoperative biliary drainage	4459	402 (61.1%)	1701 (43.9%)	<0.001
ASA-score ⩾3	4469	171 (26.0%)	947 (24.4%)	0.356
Age at diagnosis (years)*	4537	67.8 (±9.1)	65.4 (±11.4)	<0.001
Diagnosis to surgery (days)**	4485	48 (34–69)	54 (36–88)	<0.001
Body mass index (kg/m^2^)*	4318	24.7 (±4.3)	25.6 (±4.6)	<0.001
Hemoglobin (g/L)*	4338	125.8 (±16.2)	130.9 (±15.6)	<0.001
Bilirubin (μmol/L)**	4272	14 (7–30)	11 (6–23)	<0.001
C-reactive protein (mg/L)**	3962	8 (3–22)	4 (2–10)	<0.001
CA 19-9 (kE/L)**	3078	250 (39–1172)	34 (10–172)	<0.001
CEA (μmol/L)**	1234	4 (2–8)	3 (1–4)	<0.001
WBC count (×10^9^/L)**	4143	7.7 (6.2–9.6)	7.2 (6.0–8.9)	<0.001
CA 19-9-bilirubin-ratio**	3022	12.6 (2.3–74.3)	2.5 (0.9–10.2)	<0.001

ASA: American Society of Anesthesiologists; CA: cancer antigen; CEA: carcinoembryonic antigen; WBC: white blood cells.

Data are presented as absolute numbers (percentage) except where *mean (standard deviation) and **median (interquartile range).

**Fig. 2.**
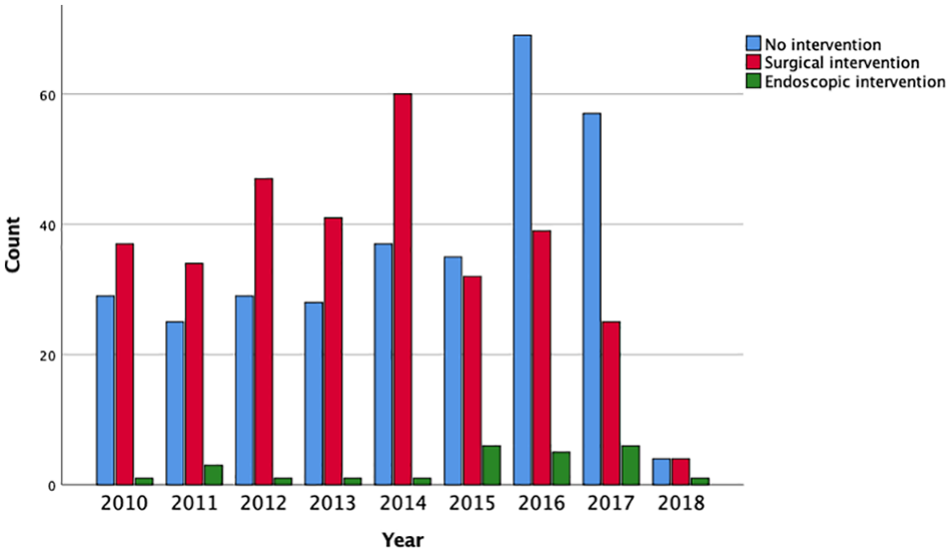
Clustered bar charts illustrating the difference in use of perioperative palliative surgery in 2010–2018.

Over the last 9 years, 335 patients with an unresectable pancreatic cancer have received palliative surgery and 26 patients have undergone endoscopic palliative surgery during the surgical exploration. In 2015, the patient group who received conservative treatment was larger than the group who underwent palliative surgery. This was the first year since the establishment of the registry as palliative surgery was performed in less than half of the patients with an unresectable tumor (Fig. 2). The same decline in palliative surgery could be seen when comparing patients who were diagnosed in 2010–2013 and 2014–August 2018 (n = 167 (58.4%) vs n = 168 (41.8%), p < 0.001).

The palliative surgical procedure performed has changed over the last years (p = 0.001, Table 3). In the first time period, double bypass was performed on 86 patients (52.1% of the patients undergoing palliative surgery). In the second time period, the same procedure was done on 55 patients (30.7%). At the same time, an increase in intraoperative endoscopic procedures could be observed, the use on stents increased from 8 patients (4.8%) in the earlier time period, compared to 21 patients (11.7%) in the later.

**Table 3.** Difference in choice of perioperative palliative intervention in 2010–2013 compared to 2014–2018.

Type of perioperative palliative surgery performed	n	2010–2013 (n = 165)	2014–2018 (n = 179)
Hepaticojejunostomy	67	29 (17.6%)	38 (21.2%)
Gastroenteroanastomosis	65	28 (17.0%)	37 (20.7%)
Hepaticojejunostomy + gastroenteroanastomosis (double bypass)	141	86 (52.1%)	55 (30.7%)
Wallstent	25	6 (3.6%)	19 (10.6%)
Wallstent + gastroenteroanastomosis	4	2 (1.2%)	2 (1.1%)
Other/unknown intervention	42	14 (8.5%)	28 (15.6%)

Data are presented as absolute numbers (percentage). The difference between the performed palliative surgery and the two time eras is statistically significant (chi-square-test, p = 0.001).

**Fig. 3.**
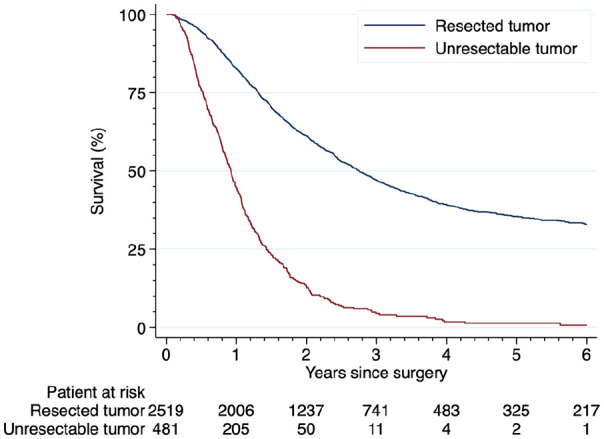
Kaplan–Meier curve illustrating the difference in survival between the patients with an unresectable tumor in comparison to the patients with a resectable tumor. The difference between the groups is statistically significant (p < 0.001).

In August 2018, 3178 patients (25.7%) were alive and 9199 patients (74.3%) were deceased. For the pancreatic and periampullary tumor group (2463 resected, 481 unresectable), 56 patients (1.9%) died within 30 days of surgery and 142 patients (4.8%) within 90 days of surgery. The 30-day mortality was higher among the unresectable patients compared to the resectable (n = 17 (3.5%) vs n = 39 (1.6%), p = 0.004). The same result was seen when analyzing 90-day mortality (n = 72 (15.0%) vs n = 70 (2.8%), p < 0.001). The survival rate was lower among the patients with an unresectable tumor compared to patients with a resectable tumor (p < 0.001) (Fig. 3).

## Discussion

In this study, we have investigated a national cohort of patients subjected to pancreatic surgery, including all diagnosis, and primarily focusing on the ones found unresectable during surgical exploration, with in-depth analysis of patients with pancreatic and periampullary tumors. Of the 4568 patients undergoing surgery, an unresectable tumor or a tumor spread was found in 658 cases. As expected, the survival rate was lower among the patients with an unresectable tumor compared to patients with a resectable tumor. More patients underwent surgical exploration and resection during the second time period, but exploration without resection was unchanged. The role of palliative surgery changed, with a decrease over the study period.

Although the risk of complications has decreased over the years, pancreatic surgery is still associated with a high perioperative mortality. The 30-day mortality after surgery ranges between 1% and 2%^9,11^, which corresponds well with our patient material (1.9%). The higher mortality rates among the unresectable group in comparison to the resectable group were expected. A high 30-day mortality was also seen in a cohort including only pancreatic cancer patients^12^. Having an unresectable tumor indicates a more disseminated disease, which should impact the expected survival negatively. As many as 15.1% of the patients with a non-resectable tumor died within 90 days in the present material including patients with pancreatic and periampullary cancer.

More patients underwent exploration and resection in the second time period, which is in line with previous findings^13,14^. Some possible reasons is that the criteria for local resectability have been widen and neoadjuvant treatment has been introduced, enabling more patients to be treated with curative intent despite extensive vascular involvement^15^. Also, the knowledge of premalignant lesions such as intraductal papillary mucinous neoplasm (IPMN) has been increasing, which could cause a rise in the amount of resections^16,17^. The reason that the proportion compared to all patients in this register was unchanged is deemed due to a progressively increased inclusion of patients, where it initially was better coverage for those who were intended for resection compared to those who were palliative already at the time of the diagnosis^18^.

Among patients who underwent surgical exploration, the proportion of patients with tumor resection did not increase. In an article including only pancreatic cancer patients, an increased resection rate was seen comparing 2009 and 2013, but still almost one-third of patients with surgical exploration for pancreatic cancer did not undergo resection^12^. During the last years, a centralization of pancreatic surgery has been completed in many countries and the sensitivity of preoperative imaging has been improving^9,12,19^. However, still exploration without resection is an important topic and problem, both for the individual patient that is subjected to unnecessary surgery with subsequent delay of palliative therapy and from a health economic perspective.

This study has highlighted a number of possible risk factors for having an unresectable pancreatic lesion. Many of these factors are known risk factors for unresectable pancreatic cancer, such as older age^20^, involuntary weight loss^21^, diabetes mellitus^22^, lower hemoglobin^23^, higher CRP^24^, and higher CA 19-9^25^. However, all registered patients did not have pancreatic or periampullary cancer. Some patients had premalignant tumors (e.g. IPMN), and some had metastases from other cancerous diseases. A smaller proportion even had benign lesions. Due to the heterogeneity in the patient material, it is not possible to make further or deeper analyzes of potential risk factors for unresectable disease. However, since surgical exploration without resection is common, with no decrease over time, a preoperative risk score may be important to improve the preoperative decision. This could be of value both for the individual patient as well as for resource allocation.

The study findings regarding the declining use of perioperative palliative surgery were expected. The results are in line with previous studies, which suggests a careful approach when a pancreatic tumor is deemed unresectable^5,6^. The findings illustrate a paradigm shift regarding perioperative palliative surgery. Patients, who previously were subjected to double-bypass surgery, are now treated more conservatively. This results in less complications, and a shorter hospital stay for the patient^6^.

While palliative surgery has decreased over time, the proportion of intraoperative endoscopic procedures, primarily with self-expanding metallic stents, has increased. These interventions are, unlike palliative surgery, usually performed only in case of a symptomatic biliary and/or gastric outlet obstruction, and can safely be performed both during surgery or postoperatively^26^. Endoscopic procedures are favorable for several reasons. They do not affect morbidity as much as palliative surgery does, and the length of the hospital stay is shorter^6^. The costs are also lower^27,28^.

The strengths of this study is the large patient material, that the registry is validated for registered data, and that the accuracy and coverage is reported annually^9^. The study has limitations inherent to analysis of registry, for example, the availability of specific data, the quality of the source data, and the number of missing data.

In conclusion, surgical exploration without resection is common in patients scheduled for pancreatic surgery. An unresectable pancreatic or periampullary tumor is associated with an adverse prognosis, including also a high risk for early mortality. The rate of completed resections did not increase, but the use of perioperative palliative surgery decreased during the study period. For the future, more studies have to be done to improve the preoperative decision regarding if the patient is resectable or not.
